# Therapeutic potential of selenium in breast cancer: targeting apoptosis and proliferation pathways

**DOI:** 10.1097/MS9.0000000000004480

**Published:** 2025-12-03

**Authors:** Emmanuel Ifeanyi Obeagu

**Affiliations:** a Department of Biomedical and Laboratory Science, Africa University, Mutare, Zimbabwe; b The Department of Molecular Medicine and Haematology, School of Pathology, Faculty of Health Sciences, University of the Witwatersrand, Johannesburg, South Africa

**Keywords:** apoptosis, breast cancer, proliferation, redox signaling, selenium

## Abstract

Selenium, an essential trace element, has garnered significant attention for its role in modulating key cellular processes in cancer biology, particularly within breast cancer pathogenesis. Emerging evidence suggests that selenium exhibits a dual role – acting as both an antioxidant and a pro-oxidant – depending on its concentration and chemical form. This dynamic behavior enables selenium to influence critical pathways associated with apoptosis and proliferation, which are often dysregulated in breast cancer. By modulating oxidative stress and redox signaling, selenium plays a unique role in maintaining the balance between cell survival and programmed cell death. Molecular studies have revealed that selenium induces apoptosis in breast cancer cells through both intrinsic and extrinsic pathways, involving mitochondrial dysfunction, caspase activation, and regulation of key genes such as p53, Bax, and Bcl-2. Simultaneously, selenium impairs tumor growth by halting the cell cycle and suppressing proliferative signals via PI3K/Akt/mTOR and MAPK pathways. These antiproliferative and pro-apoptotic actions position selenium as a promising agent for both prevention and therapy, especially when considered in combination with conventional anticancer drugs. However, selenium’s paradoxical effects at higher doses underscore the need for cautious dose optimization and personalized approaches.

## Introduction

Breast cancer remains one of the leading causes of cancer-related morbidity and mortality among women worldwide. Despite significant advancements in screening, diagnostics, and treatment, the intricate interplay of molecular pathways governing tumor progression and therapeutic resistance continues to pose challenges. Central to the pathophysiology of breast cancer is the dysregulation of cellular mechanisms that control proliferation and apoptosis. A fine-tuned balance between cell division and programmed cell death is crucial for maintaining normal tissue homeostasis, and its disruption contributes to uncontrolled tumor growth and malignancy^[1,2]^. Apoptosis, or programmed cell death, serves as a natural safeguard against cancer development by eliminating damaged or abnormal cells. In contrast, proliferation drives tissue growth and regeneration but can become pathological when unregulated. In breast cancer, the evasion of apoptosis and sustained proliferative signaling are hallmark capabilities that enable tumor cells to thrive^[3,4]^. Selenium, a trace element essential to human health, has garnered attention for its potential anticancer properties, particularly in hormone-related cancers such as breast cancer. Selenium exerts its biological effects through its incorporation into selenoproteins, many of which are involved in redox regulation and cellular defense mechanisms. The element’s ability to influence both oxidative stress and immune function makes it a compelling candidate for cancer chemoprevention and therapy^[5,6]^. The dual nature of selenium – exhibiting both antioxidant and pro-oxidant properties – underpins its unique role in modulating cell fate. At physiological levels, selenium helps maintain redox homeostasis and supports cellular function, while at supra-physiological concentrations, it can induce oxidative stress selectively in tumor cells, leading to apoptosis. This dichotomy has been exploited in experimental cancer models to selectively target cancer cells while sparing normal tissues^[7–10]^.

**HIGHLIGHTS**

- Selenium modulates key apoptotic pathways in breast cancer cells.
- Enhances tumor suppressor activity and reduces oncogene expression.
- Disrupts cancer cell proliferation and cell cycle progression.
- Synergizes with chemotherapy to improve efficacy.
- Exhibits subtype-specific effects, especially in triple-negative breast cancer.

In breast cancer cells, selenium has been shown to induce apoptosis via both the intrinsic (mitochondrial) and extrinsic (death receptor-mediated) pathways. It can activate key molecular players such as caspases, increase pro-apoptotic proteins like Bax, and inhibit antiapoptotic proteins like Bcl-2. Moreover, selenium influences the tumor suppressor protein p53, further contributing to its apoptotic effects. These mechanisms suggest that selenium may help overcome apoptosis resistance, a common barrier to effective cancer therapy^[11,12]^. On the other hand, selenium also exerts antiproliferative effects by modulating signaling pathways that govern cell cycle progression. The inhibition of proliferative cascades such as the PI3K/Akt/mTOR and MAPK pathways has been observed in selenium-treated breast cancer cells. Furthermore, selenium has been shown to downregulate cyclins and cyclin-dependent kinases, effectively arresting the cell cycle at various checkpoints. These properties make selenium a potential candidate for controlling tumor growth and metastasis^[13,14]^. While preclinical findings are promising, translating these effects into clinical outcomes has proven complex. The efficacy of selenium appears to be influenced by multiple factors, including its chemical form (organic vs inorganic), dosage, baseline selenium status of the patient, and genetic background. Some clinical trials have reported protective effects of selenium supplementation, while others have shown no benefit or even potential harm, underscoring the need for a personalized approach to selenium-based therapy^[15–17]^.

## Aim

The aim of this review is to explore the multifaceted role of selenium in breast cancer biology, focusing on its ability to modulate apoptosis and proliferation in breast cancer cells.

## Methods

This narrative review was conducted to synthesize current knowledge on the therapeutic potential of selenium in breast cancer, with a focus on its regulatory effects on apoptosis and proliferation pathways. A comprehensive literature search was performed using PubMed, Scopus, and Web of Science databases. Key search terms included *“selenium,” “breast cancer,” “apoptosis,” “proliferation,” “oxidative stress,” “selenoproteins,” “chemoprevention,”* and *“therapeutic supplementation.”* Articles published between 2000 and 2025 were considered, including preclinical studies, clinical trials, and mechanistic investigations. Both organic [selenomethionine, methylselenocysteine, methylseleninic acid (MSA)] and inorganic forms (selenite, selenate) of selenium were included to capture their diverse biological effects. Studies addressing apoptotic signaling, cell cycle regulation, tumor proliferation, oxidative stress modulation, and clinical supplementation outcomes were prioritized.

Evidence from the retrieved literature was synthesized narratively to provide a conceptual and mechanistic understanding rather than a statistical meta-analysis. Particular attention was given to integrating data across cellular models, animal studies, and human clinical trials, highlighting translational implications. Where relevant, quantitative findings such as tumor growth inhibition percentages, apoptosis induction rates, and serum selenium concentrations were extracted and discussed to strengthen the evidence-based context. This narrative approach allowed for flexible synthesis of mechanistic insights and clinical applications, emphasizing emerging trends and translational potential of selenium in breast cancer management. Limitations of the included literature, including heterogeneity in study designs, selenium forms, and dosage regimens, were acknowledged to provide a balanced perspective.

## Selenium and apoptosis in breast cancer

The process of apoptosis, or programmed cell death, serves as a critical barrier against cancer development by eliminating damaged, abnormal, or potentially malignant cells. In breast cancer, however, this self-destruct mechanism is frequently compromised, allowing for the survival and expansion of aberrant cells. Among the various agents explored for their potential to restore apoptotic signaling in malignant tissues, selenium has emerged as a particularly intriguing candidate^[17]^. Selenium’s pro-apoptotic action in breast cancer is multifaceted, involving a fine interplay of redox modulation, mitochondrial destabilization, and transcriptional reprogramming. At the core of selenium-induced apoptosis lies its capacity to disturb the redox homeostasis within cancer cells. Unlike normal cells, which maintain a relatively stable redox environment, breast cancer cells often exist in a state of heightened oxidative stress. Selenium compounds, particularly in their inorganic forms like sodium selenite, exploit this vulnerability by amplifying reactive oxygen species (ROS) production. The resulting oxidative burst overwhelms cellular antioxidant defenses, initiating a cascade of events that lead to mitochondrial dysfunction and cell death^[18]^. One of the key consequences of selenium-mediated ROS generation is the permeabilization of the mitochondrial membrane. This event disrupts the mitochondrial membrane potential and facilitates the release of pro-apoptotic factors such as cytochrome c into the cytosol. This release sets off the intrinsic apoptotic pathway, activating caspase-9 and subsequently caspase-3, the executioners of apoptosis. Several studies have confirmed this cascade in breast cancer cell lines treated with selenium, particularly highlighting the critical role of mitochondrial destabilization^[19]^.

Selenium also influences the expression of proteins that tightly regulate the mitochondrial apoptotic pathway. For instance, it upregulates pro-apoptotic members of the Bcl-2 family, such as Bax and Bak, while simultaneously downregulating antiapoptotic proteins like Bcl-2 and Bcl-xL. This shift in the Bax/Bcl-2 ratio further tips the balance toward apoptosis, making breast cancer cells more susceptible to programmed cell death^[20]^. In addition to its effects on the intrinsic pathway, selenium has been shown to engage the extrinsic or death receptor-mediated apoptotic pathway. By upregulating surface expression of death receptors such as Fas and TRAIL-R1/2, selenium sensitizes breast cancer cells to apoptosis initiated through ligand–receptor interactions. This property is particularly promising for combinational strategies involving TRAIL-based therapeutics, which have shown potential in inducing tumor-selective apoptosis^[21]^. Interestingly, selenium’s pro-apoptotic activity is not uniform across all breast cancer subtypes. Its effects are often more pronounced in triple-negative breast cancer (TNBC) cells, which lack estrogen, progesterone, and HER2 receptors and are typically more aggressive and resistant to conventional therapies. In these cells, selenium’s capacity to induce oxidative stress and activate apoptotic pathways appears to be especially effective, suggesting a potential therapeutic niche^[22]^.

The chemical form of selenium also plays a decisive role in dictating its apoptotic effects. While inorganic forms like selenite tend to induce apoptosis via oxidative stress mechanisms, organic selenium compounds such as methylselenocysteine often act through redox-independent mechanisms, including modulation of gene expression and inhibition of oncogenic signaling. Regardless of the form, however, a unifying theme emerges: selenium’s ability to push malignant cells toward apoptosis while sparing – or even protecting – normal cells, provided the dose is carefully controlled^[23]^. This dual selectivity highlights both the promise and the challenge of selenium-based interventions. The same pro-oxidant activity that renders selenium cytotoxic to cancer cells can also become harmful to normal tissues if dosing thresholds are surpassed. As such, the therapeutic utility of selenium in breast cancer hinges on optimizing its delivery and exploiting tumor-specific vulnerabilities (Table 1)^[24]^.

**Table 1:** Selenium and apoptosis in breast cancer

Selenium compound	Breast cancer cell line(s)	Apoptotic mechanism	Key findings
Sodium selenite	MCF-7, MDA-MB-231	Reactive oxygen species (ROS) generation, mitochondrial membrane depolarization, cytochrome c release, caspase-3/9 activation	Induced dose-dependent apoptosis; increased Bax/Bcl-2 ratio; selective cytotoxicity in cancer cells
Methylselenocysteine	MCF-7	Cell cycle arrest at G1, upregulation of p21^Cip1^ and pro-apoptotic signaling	Suppressed proliferation and triggered apoptosis without affecting normal breast epithelial cells
Selenomethionine	T47D, MCF-7	Downregulation of Bcl-2, activation of caspases, DNA fragmentation	Enhanced apoptosis, especially when combined with chemotherapy
Selenium nanoparticles	MDA-MB-231, 4T1	Enhanced ROS production, loss of mitochondrial membrane potential, caspase cascade activation	Greater apoptosis induction than sodium selenite; lower systemic toxicity in *in vivo* models
Se-enriched yeast extract	MCF-7	Modulation of Fas/FasL death receptor pathway	Promoted extrinsic apoptosis; potential for use in dietary-based chemoprevention
Sodium selenite + TRAIL	MDA-MB-231	Sensitization to TRAIL-mediated apoptosis via upregulation of death receptors (DR4/DR5) and caspase-8 activation	Synergistic apoptotic effect; potential to overcome TRAIL resistance

## Crosstalk between apoptotic pathways in breast cancer and selenium regulation

**Figure 1.**
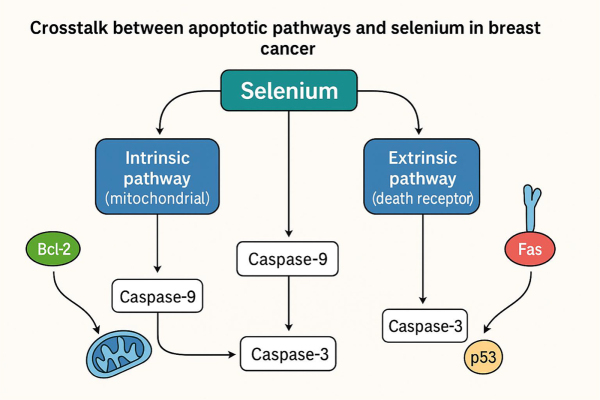
Crosstalk between apoptotic pathways and selenium in breast cancer.

Apoptosis, or programmed cell death, is a tightly regulated process essential for maintaining tissue homeostasis. In breast cancer, dysregulation of apoptotic pathways contributes to uncontrolled proliferation, treatment resistance, and tumor progression. Selenium has been shown to modulate apoptosis through both intrinsic (mitochondrial) and extrinsic (death receptor) pathways, and emerging evidence highlights significant crosstalk between these two routes, which may amplify tumor cell susceptibility to selenium-induced cytotoxicity (Fig. 1) ^[17,18]^.

### Intrinsic (mitochondrial) pathway

The intrinsic pathway is initiated by cellular stress signals such as oxidative stress, DNA damage, or oncogene activation. Selenium compounds, particularly MSA and sodium selenite, induce mitochondrial membrane permeabilization, leading to cytochrome c release into the cytoplasm. This event triggers caspase-9 activation, which in turn activates effector caspase-3 and caspase-7, culminating in apoptosis. Selenium also modulates the Bcl-2 family proteins, promoting pro-apoptotic members (Bax, Bak) and suppressing antiapoptotic members (Bcl-2, Bcl-xL), thereby lowering the apoptotic threshold in tumor cells^[19,20]^.

### Extrinsic (death receptor) pathway

The extrinsic pathway is mediated by cell surface death receptors such as Fas (CD95) and TRAIL-R (DR4/DR5). Upon ligand binding, these receptors recruit adaptor proteins and activate caspase-8, which can directly cleave effector caspases or engage mitochondrial amplification loops via Bid cleavage, linking extrinsic signals to the intrinsic pathway. Selenium has been shown to upregulate Fas and TRAIL receptor expression in breast cancer cells, enhancing sensitivity to receptor-mediated apoptosis without affecting normal epithelial cells^[21,22]^.

### Crosstalk between pathways

The bidirectional communication between intrinsic and extrinsic pathways allows amplification of apoptotic signaling. Selenium facilitates this crosstalk by:

- Enhancing Bid cleavage downstream of caspase-8 activation, which triggers mitochondrial cytochrome c release.
- Modulating ROS levels, which simultaneously activates stress-induced intrinsic signaling and sensitizes death receptor pathways.
- Augmenting p53 activity, thereby coordinating transcriptional upregulation of pro-apoptotic genes involved in both pathways.

This integration ensures that sub-threshold activation in one pathway can be amplified through the other, increasing the likelihood of cancer cell death while sparing normal cells. Preclinical studies suggest that selenium-induced crosstalk is particularly pronounced in triple-negative breast cancer (TNBC), where high oxidative stress and lack of hormonal regulation render tumor cells more susceptible to coordinated apoptotic activation (Table 2) ^[23,24]^.

**Table 2:** Molecular mechanisms of selenium-induced apoptosis

Molecular target/pathway	Effect of selenium	Functional outcome	Notes
Reactive oxygen species (ROS)	Increased ROS generation leading to oxidative stress	Mitochondrial damage and initiation of intrinsic apoptosis	Critical for selective cancer cell death
Mitochondrial membrane potential (Δψm)	Disruption and depolarization	Release of cytochrome c into cytoplasm	Activates caspase-9 and downstream caspases
Bcl-2 family proteins	Upregulation of pro-apoptotic Bax; downregulation of antiapoptotic Bcl-2	Promotion of mitochondrial outer membrane permeabilization	Shifts balance toward apoptosis
Caspases (3, 8, 9)	Activation of initiator (caspase-8, caspase-9) and executioner caspases (caspase-3)	Proteolytic cleavage of key substrates, apoptosis execution	Both intrinsic and extrinsic pathways involved
Death receptors (Fas, DR4, DR5)	Upregulation and enhanced sensitivity to ligand binding (e.g., FasL, TRAIL)	Initiation of extrinsic apoptosis pathway	Sensitizes cells to death receptor-mediated apoptosis
p53 tumor suppressor	Stabilization and activation	Transcriptional activation of pro-apoptotic genes	Enhances apoptosis, especially in p53 wild-type cells
NF-κB signaling	Inhibition leading to reduced expression of antiapoptotic genes	Promotes apoptosis by decreasing survival signals	Contributes to chemosensitization
Endoplasmic reticulum (ER) stress	Induction of unfolded protein response pathways	Activation of ER-stress-mediated apoptosis	Observed with high selenium doses

## Selenium and proliferation control in breast cancer

The proliferative capacity of cancer cells underpins their aggressive growth and resistance to treatment, and in breast cancer, the loss of regulatory control over cell division plays a pivotal role in tumor progression and metastasis. At the heart of this dysregulation lies the disruption of key signaling pathways and checkpoints that normally govern the cell cycle. Intriguingly, selenium – an essential micronutrient with recognized antioxidant and anticarcinogenic properties – has emerged as a promising modulator of these proliferative signals, offering a potential avenue for therapeutic intervention^[25]^. Selenium exerts its influence on proliferation through multiple, intersecting mechanisms, many of which converge on the cell cycle machinery. In breast cancer models, both *in vitro* and *in vivo*, selenium compounds have demonstrated a capacity to halt cell cycle progression at critical junctures – most notably in the G1 and G2/M phases. This cell cycle arrest is not merely a by-product of cellular stress; rather, it reflects targeted modulation of cell cycle regulators. Selenium has been shown to upregulate the expression of cyclin-dependent kinase inhibitors such as p21^Cip1/Waf1 and p27^Kip1 – key molecules that act as molecular brakes on cell cycle advancement. Simultaneously, it downregulates cyclins and CDKs, including cyclin D1 and CDK4/6, which are frequently overexpressed in breast cancer and are essential drivers of G1 to S phase transition^[26]^.

Beyond direct cell cycle regulation, selenium also interacts with proliferative signaling cascades, notably the PI3K/Akt and MAPK/ERK pathways. These pathways are commonly dysregulated in breast cancer and are intimately involved in promoting cell survival, growth, and resistance to apoptosis. Selenium compounds have been found to inhibit the activation of these kinases, leading to diminished mitogenic signaling and suppressed cell proliferation. By attenuating these oncogenic pathways, selenium not only disrupts the growth advantage of tumor cells but may also sensitize them to conventional therapies^[27]^. Importantly, the effects of selenium on proliferation appear to be context-dependent, influenced by its chemical form, concentration, and the molecular profile of the tumor. For instance, organic selenium species such as methylselenocysteine have demonstrated more potent antiproliferative effects in some breast cancer models compared to their inorganic counterparts. Notably, selenium exhibits selective cytotoxicity toward malignant breast cells while preserving the viability of normal epithelial cells, a property that underscores its therapeutic potential^[28]^. The interplay between selenium and estrogen receptor (ER) signaling offers another layer of complexity. In hormone-responsive breast cancers, estrogen promotes proliferation through genomic and non-genomic mechanisms. Selenium has been shown to interfere with estrogen-mediated signaling, potentially by downregulating ER expression or disrupting ER-driven transcriptional programs. This raises the possibility of selenium enhancing the efficacy of endocrine therapies or serving as a chemopreventive agent in ER-positive disease (Table 3)^[29]^.

**Table 3:** Selenium and proliferation control in breast cancer

Selenium compound	Breast cancer cell line(s)	Mechanism of proliferation inhibition	Key findings
Sodium selenite	MCF-7, MDA-MB-231	Induces G2/M cell cycle arrest via upregulation of p21 and downregulation of cyclin B1	Significant reduction in proliferation; cell cycle arrest at G2/M checkpoint
Methylselenocysteine	MCF-7	G1 phase arrest mediated by increased p27^Kip1^ and decreased cyclin D1	Suppressed DNA synthesis and cell cycle progression
Selenomethionine	T47D	Inhibition of PI3K/Akt signaling pathway	Reduced cell viability and proliferation; enhanced sensitivity to apoptosis
Selenium nanoparticles	MDA-MB-231	MAPK/ERK pathway inhibition leading to decreased cyclin D1 expression	Dose-dependent inhibition of proliferation with minimal toxicity
Se-enriched yeast extract	MCF-7	Modulates estrogen receptor signaling and downstream proliferative genes	Decreased ER-mediated transcription; reduced hormone-driven proliferation

## Selenium’s antiproliferative effects in breast cancer

The interplay between unchecked cellular proliferation and tumor progression is a defining hallmark of breast cancer biology. Amidst the myriad of molecular regulators and environmental influences, selenium – an essential trace element – has emerged as a notable modulator of cancer cell proliferation. Beyond its well-known antioxidant properties, selenium exhibits a profound ability to interfere with the proliferative machinery of breast cancer cells, contributing to its growing recognition as a potential therapeutic adjunct in oncologic care^[25,26]^. At the cellular level, selenium’s antiproliferative effects stem from its capacity to disrupt critical signaling pathways that fuel cell growth and survival. Among the most targeted of these is the PI3K/Akt/mTOR axis, a central conduit of mitogenic signaling that is frequently hyperactivated in breast cancer. Selenium compounds, particularly selenomethionine and MSA, have been shown to inhibit this pathway, resulting in decreased cyclin D1 expression and subsequent arrest of the cell cycle at the G1/S checkpoint. This interruption in cell cycle progression prevents DNA replication and effectively halts the expansion of malignant cell populations^[27–29]^. Moreover, selenium modulates the expression and activity of cyclin-dependent kinases (CDKs) and their inhibitors. It downregulates CDK2 and CDK4, key drivers of cell cycle progression, while simultaneously upregulating CDK inhibitors like p21^Cip1^ and p27^Kip1^. The result is a well-orchestrated deceleration of the cell cycle, shifting the balance toward quiescence and away from unchecked division. This effect is particularly pronounced in hormone receptor-positive breast cancer cells, where selenium’s interference with ER signaling further compounds its growth-inhibitory potential^[30]^.

Another mechanism through which selenium exerts its antiproliferative influence is by disrupting the transcriptional activity of oncogenic transcription factors such as c-Myc and NF-κB. These factors are central to the transcription of genes that promote cellular replication and survival. Selenium downregulates c-Myc expression, thereby limiting the transcription of growth-promoting genes, and inhibits NF-κB activation, reducing the expression of pro-inflammatory cytokines and growth factors that contribute to a pro-proliferative tumor microenvironment^[31,32]^. Notably, selenium’s antiproliferative effects are not limited to direct inhibition of cancer cell signaling. It also exerts epigenetic influences that alter gene expression patterns in a way that disfavors proliferation. Selenium has been found to induce DNA methylation changes and histone modifications that repress oncogene expression while activating tumor suppressor genes. These modifications contribute to a more differentiated, less aggressive cellular phenotype – one that is less capable of sustained proliferation^[33]^. Furthermore, selenium’s role in modulating angiogenesis indirectly influences tumor cell proliferation. By downregulating pro-angiogenic factors like vascular endothelial growth factor (VEGF), selenium limits the formation of new blood vessels needed to supply oxygen and nutrients to rapidly growing tumor tissues. This antiangiogenic effect starves the tumor microenvironment, restricting its capacity to support continued proliferation and metastatic spread^[34]^. *In vitro* and *in vivo* studies provide compelling support for selenium’s antiproliferative role. Cell culture experiments reveal dose-dependent reductions in breast cancer cell proliferation following selenium treatment, while animal models exhibit slowed tumor growth and reduced tumor burden with selenium supplementation. Importantly, these effects are observed at selenium concentrations that are non-toxic to normal cells, highlighting selenium’s therapeutic selectivity and potential clinical utility (Table 4)^[35]^.

**Table 4:** Selenium’s antiproliferative effects in breast cancer

Effect	Mechanism	Outcome	Example selenium compound(s)
Cell cycle arrest	Upregulation of cyclin-dependent kinase inhibitors (p21^Cip1^, p27^Kip1^) and downregulation of cyclins (D1, B1)	Arrest at G1 or G2/M phases, halting cell division	Methylselenocysteine, sodium selenite
Inhibition of mitogenic signaling	Suppression of PI3K/Akt and MAPK/ERK pathways	Reduced proliferation and survival signaling	Selenomethionine, Selenium nanoparticles
Hormone receptor modulation	Downregulation of estrogen receptor expression and inhibition of ER-mediated gene transcription	Reduced hormone-driven proliferation	Se-enriched yeast extract
Induction of senescence	Activation of tumor suppressor pathways and DNA damage response	Permanent growth arrest	Sodium selenite
Epigenetic regulation	Modulation of histone acetylation and DNA methylation affecting oncogene expression	Long-term suppression of proliferation	Methylselenocysteine

## Dose-dependent effects and the selenium paradox in breast cancer

One of the most intriguing aspects of selenium’s role in breast cancer biology lies in its dose-dependent duality – often referred to as the “selenium paradox.” This phenomenon captures the essence of selenium as both a guardian of cellular integrity and a potential agent of cytotoxicity, depending on the context and concentration^[36]^. At physiological levels, selenium plays a vital role in maintaining cellular homeostasis. Incorporated into selenoproteins such as glutathione peroxidases and thioredoxin reductases, it contributes to redox regulation, DNA synthesis, and protection against oxidative stress. In this capacity, selenium supports normal cell function, bolsters antioxidant defenses, and may even offer a protective effect against the initial stages of carcinogenesis. Several epidemiological studies have suggested an inverse correlation between selenium status and breast cancer incidence, particularly in populations with marginal selenium intake. In these settings, selenium acts as a stabilizer – preventing DNA damage, modulating inflammation, and preserving the delicate equilibrium between cell proliferation and death^[37]^.

However, as selenium concentrations increase beyond physiological thresholds, its behavior shifts dramatically. In breast cancer cells, supra-nutritional or pharmacological doses of selenium – particularly in the form of inorganic selenium compounds such as sodium selenite – induce the generation of ROS, disrupt mitochondrial membrane potential, and activate intrinsic apoptotic pathways. These cytotoxic effects are largely selective to cancer cells, exploiting their heightened oxidative stress and altered redox capacity. Importantly, the oxidative burden imposed by high-dose selenium overwhelms antioxidant defenses, triggering apoptosis through mechanisms involving caspase activation, DNA fragmentation, and modulation of Bcl-2 family proteins^[38]^. This biphasic response – antioxidant and protective at low doses, pro-oxidant and cytotoxic at high doses – underscores the complexity of selenium’s role in breast cancer treatment. It is a paradox that reflects the fine line between chemoprevention and chemotherapy. While low-dose selenium may support general health and possibly reduce cancer risk, higher doses are being investigated for their therapeutic potential in selectively eliminating malignant cells. The form of selenium is also crucial; organic selenium compounds like selenomethionine tend to exhibit milder, slower biological effects compared to more reactive inorganic forms (Table 5)^[39]^.

**Table 5:** Dose-dependent effects and the selenium paradox in breast cancer

Dose range	Biological effect	Cellular mechanism	Clinical implications	Example selenium forms
Low dose (nutritional level)	Antioxidant activity; supports redox homeostasis	Enhanced activity of selenoproteins (GPxs, TrxRs); reduced oxidative stress	Maintains normal cell function; may aid cancer prevention	Selenomethionine, Se-enriched yeast
Moderate dose	Mild pro-oxidant effect; modulation of cell signaling	Activation of redox-sensitive signaling pathways; mild reactive oxygen species (ROS) generation	Potential to sensitize cancer cells to apoptosis and therapies	Methylselenocysteine, sodium selenite
High dose (pharmacological)	Cytotoxicity through oxidative stress; apoptosis induction	Excessive ROS generation; mitochondrial dysfunction; DNA damage	Selective killing of cancer cells; risk of toxicity in normal tissues	Sodium selenite, selenium nanoparticles
Excessive dose (toxic level)	Cellular damage; systemic toxicity	Uncontrolled oxidative stress; disruption of multiple cellular systems	Risk of selenium poisoning; contraindicated in therapy	Various forms

## The selenium paradox in breast cancer

The therapeutic intrigue surrounding selenium in breast cancer lies not just in its biological potential but also in the complex interplay between its form, dosage, and physiological impact – a phenomenon often referred to as the “selenium paradox.” This paradox highlights the fine line between selenium’s role as a chemopreventive agent and its capacity to become ineffective or even harmful at inappropriate doses or in unsuitable forms. As researchers strive to harness selenium’s anticancer effects, unraveling this paradox becomes essential to developing safe and effective interventions^[36,37]^. Selenium exists in various chemical forms, each with distinct metabolic pathways and bioactivities. The most commonly studied forms in cancer research include organic compounds such as selenomethionine and MSA, and inorganic salts like sodium selenite and selenate. These forms differ in their absorption, tissue distribution, and ability to generate active selenium metabolites, such as methylselenol, which is believed to be the primary mediator of selenium’s anticancer effects. While selenomethionine integrates into general protein synthesis and acts as a selenium reservoir, MSA more directly and rapidly releases methylselenol, exerting potent effects on cell cycle arrest, apoptosis, and redox modulation. This variance in activity highlights why form matters just as much as dose in determining selenium’s impact on breast cancer cells^[37–39]^.

The paradox becomes even more pronounced when dosage is considered. Selenium operates within a narrow therapeutic window – deficiency compromises antioxidant defenses and immune function, whereas excess leads to selenosis, a toxic state marked by gastrointestinal disturbances, hair and nail loss, and in extreme cases, neurological damage. In the context of cancer, supranutritional doses of selenium have shown promise in experimental settings, particularly in inhibiting proliferation and inducing apoptosis in breast cancer cells. However, clinical translation is challenging. Epidemiological and interventional studies, such as the SELECT trial, have yielded mixed outcomes – some suggesting a protective role of selenium supplementation in cancer prevention, while others show no benefit or even a potential increase in cancer risk, particularly when baseline selenium levels are already adequate^[40,41]^. This duality has sparked debate within the scientific community, prompting a re-evaluation of one-size-fits-all supplementation strategies. Individual selenium status, influenced by dietary intake, genetic polymorphisms in selenoprotein genes, and regional soil selenium levels, significantly affects how the body metabolizes and responds to selenium. For instance, individuals with lower baseline selenium may derive protective benefits from supplementation, whereas those with adequate or high selenium stores may experience no added benefit or increased oxidative stress due to excessive selenium intake. This interindividual variability further complicates efforts to define an optimal therapeutic window^[42]^.

Moreover, the timing of selenium administration plays a critical role in its paradoxical behavior. Preventive supplementation in early stages or before malignancy arises may bolster antioxidant capacity and genomic stability, reducing the likelihood of tumorigenesis. In contrast, high-dose selenium administered during advanced stages of breast cancer may have different effects depending on the tumor’s redox state and resistance mechanisms. The same selenium compound that induces apoptosis in one cellular context may foster survival in another by upregulating detoxification enzymes or interfering with chemotherapeutic drugs^[43]^. Thus, the selenium paradox in breast cancer is not merely a question of “how much” but also “which form,” “when,” and “for whom.” Future clinical strategies must embrace precision nutrition principles, integrating selenium biomarkers, genetic screening, and molecular tumor profiling to tailor interventions. Rather than blanket recommendations, selenium’s use in breast cancer therapy may require stratified approaches that respect its dual nature – an essential micronutrient with both life-saving and potentially deleterious properties, depending on the context^[44]^.

## Selenium and hormone-responsive breast cancer

Breast cancer is a molecularly diverse disease, with ER-positive (ER +) subtypes representing the most prevalent form. These tumors are characterized by their dependence on estrogen signaling for growth and survival, rendering them responsive to endocrine therapies such as tamoxifen, aromatase inhibitors, and fulvestrant. However, resistance to endocrine treatment – whether intrinsic or acquired – poses a significant clinical challenge, underscoring the need for adjunctive strategies that can modulate hormone signaling and sensitize tumor cells to therapy. In this context, selenium has emerged as a compound of interest, given its unique redox-modulating and antiproliferative properties^[45].^ Selenium’s influence on hormone-responsive breast cancer appears to be multifaceted. Experimental evidence suggests that selenium compounds, particularly organic forms such as methylselenocysteine and selenomethionine, can downregulate ER alpha (ERα) expression in breast cancer cells. This suppression occurs, at least in part, through selenium-induced modulation of transcriptional regulators and co-activators involved in ER signaling. Furthermore, selenium may alter the phosphorylation status of ERα, thereby influencing its ligand-binding affinity and transcriptional activity. Such modulation of ERα function has the potential to attenuate estrogen-mediated proliferative signals, especially in ER + breast cancer subtypes^[46]^.

Beyond direct effects on ERs, selenium exerts broader regulatory control over signaling pathways that intersect with the endocrine axis. The phosphoinositide 3-kinase (PI3K)/Akt and mitogen-activated protein kinase (MAPK) pathways, both implicated in endocrine resistance, are susceptible to redox-sensitive modulation by selenium. By attenuating these survival pathways, selenium may restore apoptotic sensitivity in hormone-responsive tumors that have developed resistance to conventional therapies. Notably, selenium-induced oxidative stress, when maintained within a therapeutic threshold, appears to selectively sensitize cancer cells without compromising normal tissue integrity – an attribute of significant translational relevance^[47]^. The interplay between selenium and endocrine therapy is further illustrated in combination studies, where selenium has been shown to enhance the efficacy of tamoxifen and other antiestrogenic agents. Synergistic effects include greater induction of apoptosis, more pronounced cell cycle arrest, and decreased clonogenic survival of breast cancer cells. These observations raise the possibility that selenium may potentiate therapeutic responses in patients with suboptimal or diminishing endocrine sensitivity^[48]^.

## Selenium in precision oncology in breast cancer

As the era of precision oncology unfolds, the focus in cancer treatment is shifting from generalized therapeutic strategies to personalized interventions tailored to the individual’s unique molecular, genetic, and environmental profile. Within this context, selenium – a trace element long recognized for its antioxidant and chemopreventive properties – is garnering renewed interest. Selenium’s dualistic behavior, acting as both a protective and potentially toxic agent, makes it a prime candidate for precision modulation in breast cancer care. The future of selenium in oncology may no longer lie in population-wide supplementation but in its integration into personalized treatment plans that reflect each patient’s specific needs, risks, and biological responses^[45,46]^. One of the central pillars of precision oncology is understanding interindividual variability, and selenium metabolism is deeply influenced by genetic and epigenetic factors. Polymorphisms in genes encoding selenoproteins, such as GPX1, SEPP1, and SELENOP, can significantly affect the synthesis, transport, and antioxidant function of selenium in the body. In breast cancer patients, these variations have been linked to differences in tumor aggressiveness, response to therapy, and overall prognosis. For instance, certain variants of the GPX1 gene are associated with diminished antioxidant defense and increased oxidative DNA damage, thereby influencing susceptibility to carcinogenesis. Precision oncology offers the potential to screen for such polymorphisms and determine whether selenium supplementation would be beneficial, neutral, or harmful for a particular patient^[47,48]^. Moreover, selenium’s role in modulating molecular signaling pathways aligns closely with the mechanistic targets of modern breast cancer therapies. Selenium metabolites have been shown to interact with key regulators of cell proliferation and apoptosis, including p53, caspases, and PI3K/Akt signaling cascades. Importantly, some of these pathways are already being targeted by FDA-approved therapies in breast cancer, such as PI3K inhibitors and CDK4/6 inhibitors. Selenium, when administered in the appropriate form and concentration, may augment these targeted treatments by enhancing tumor suppression mechanisms or sensitizing cancer cells to chemotherapeutic agents. However, the success of such synergistic strategies hinges on precise molecular profiling to avoid adverse interactions and maximize therapeutic gain^[49]^.

Advancements in high-throughput technologies – such as transcriptomics, proteomics, and metabolomics – are making it increasingly feasible to assess a patient’s selenium status, selenoprotein expression, and tumor redox profile in real time. These technologies can help clinicians identify which patients might benefit from selenium-enhanced treatment regimens or who may require dose adjustments based on metabolic efficiency. For example, tumors with high oxidative stress signatures may be more susceptible to selenium-induced apoptosis, while tumors with robust antioxidant systems may resist such effects or even exploit selenium to support survival^[47]^. Furthermore, regional and dietary factors influence selenium exposure and efficacy, emphasizing the need for geographic and nutritional precision. Soil selenium content varies widely around the globe, affecting food selenium levels and, ultimately, human selenium status. Patients in selenium-deficient regions may present with suboptimal immune and antioxidant responses, potentially impacting treatment outcomes. Personalized supplementation, guided by serum selenium measurements and dietary assessments, could restore balance and contribute to more favorable prognoses^[48]^. In clinical practice, integrating selenium into precision oncology will require a multidisciplinary approach involving oncologists, nutritionists, molecular pathologists, and pharmacogenomic specialists. This integration could manifest in selenium-inclusive decision support tools, individualized supplementation protocols, and companion diagnostics that flag relevant genetic or metabolic profiles. While current clinical trials are still catching up with these conceptual advances, the groundwork is being laid for selenium to transition from a generalized nutritional supplement to a precisely targeted adjunct in breast cancer therapy^[49]^.

## Clinical implications in breast cancer

The multifaceted biological activities of selenium in modulating cellular redox balance, apoptosis, and proliferation offer compelling implications for the clinical management of breast cancer. As therapeutic resistance and tumor heterogeneity continue to impede curative outcomes, selenium-based strategies provide a potentially valuable adjunct to conventional treatments. One of the most clinically significant features of selenium is its capacity to selectively induce apoptosis in malignant cells while sparing normal tissue – a phenomenon largely attributed to the differential redox environment between cancerous and non-cancerous cells. This selectivity holds particular promise for reducing the off-target toxicities commonly associated with chemotherapy and radiation therapy. Preclinical studies have demonstrated that selenium compounds, especially in their organoselenium forms such as methylselenocysteine and seleno-L-methionine, can sensitize breast cancer cells to standard chemotherapeutic agents by enhancing oxidative stress and disrupting survival signaling pathways like PI3K/Akt and MAPK/ERK^[50]^. Furthermore, selenium’s ability to induce cell cycle arrest and suppress tumor-promoting inflammation suggests a role in preventing tumor progression and recurrence. In hormone receptor-positive breast cancer, selenium has been shown to modulate ER signaling and inhibit estrogen-driven proliferation. This opens avenues for its incorporation into endocrine therapy regimens, potentially improving responsiveness to agents like tamoxifen or aromatase inhibitors while mitigating resistance mechanisms^[51]^.

Clinical trials evaluating selenium supplementation in cancer prevention have yielded inconsistent results, in part due to variations in baseline selenium status, forms of selenium used, and study populations. Notably, findings from the Selenium and Vitamin E Cancer Prevention Trial (SELECT) highlight the importance of personalized and precision-based approaches in selenium intervention. Rather than indiscriminate supplementation, future clinical strategies may benefit from biomarker-guided administration, considering genetic polymorphisms in selenoprotein genes, tumor molecular profiles, and systemic selenium levels^[52]^. The development of targeted delivery systems, such as selenium-conjugated nanoparticles, is another emerging area with clinical relevance. These platforms aim to enhance selenium’s bioavailability and tumor specificity, thereby maximizing therapeutic efficacy while minimizing systemic toxicity. Additionally, ongoing investigations into the immunomodulatory effects of selenium suggest that it may also have a synergistic role in enhancing the efficacy of immune checkpoint inhibitors, particularly in TNBC, where immunotherapy is gaining traction^[53]^.

## Therapeutic potential of selenium in breast cancer

The therapeutic potential of selenium in breast cancer has been supported by both experimental and early-phase clinical studies demonstrating its ability to modulate apoptosis, oxidative stress, and cell proliferation. However, the clinical translation of these findings hinges on addressing four fundamental questions: *how much, which form, when*, and *for whom* selenium should be used. Understanding these dimensions is essential for optimizing therapeutic benefit while minimizing toxicity.

### How much? (dosage ranges and safety window)

The biological activity of selenium follows a dose-dependent, U-shaped curve – protective at physiological levels but potentially harmful when excessive. The recommended dietary intake for adults is approximately 55 μg/day, while therapeutic studies in oncology have explored doses ranging from 100 to 400 μg/day. Clinical safety data suggest that doses above 800 μg/day may induce selenosis, characterized by gastrointestinal upset, hair loss, and neuropathy.Experimental evidence indicates that selenium’s anti-tumorigenic effects are achieved at serum concentrations between 120 and 160 μg/L, a range sufficient to activate selenoenzymes such as glutathione peroxidase and thioredoxin reductase, which mitigate oxidative DNA damage in breast tissue. Thus, therapeutic application requires a carefully titrated supplementation strategy to maintain efficacy without exceeding the narrow toxicity threshold^[53]^.

### Which form? (bioavailable organic and inorganic selenium)

Selenium exists in multiple chemical forms with distinct bioavailability and metabolic fates. Organic forms – such as selenomethionine (SeMet) and methylselenocysteine (MSC) – are efficiently absorbed and incorporated into proteins, providing sustained systemic activity. Inorganic forms – including sodium selenite and selenate – exhibit higher reactivity and can induce oxidative stress selectively in cancer cells, promoting apoptosis.Among these, MSA has gained prominence in experimental oncology for its potent pro-apoptotic and antiproliferative effects through redox modulation and activation of p53-mediated pathways. Meanwhile, selenium nanoparticles (SeNPs) have emerged as next-generation formulations that improve bioavailability, enhance tumor targeting, and reduce systemic toxicity. The therapeutic form of selenium should therefore be tailored to the intended mechanism – antioxidant support versus direct tumor cytotoxicity^[54]^.

### When? (timing of supplementation and disease stage)

The timing of selenium administration profoundly influences its therapeutic outcomes. In early-stage or pre-malignant breast lesions, selenium acts primarily as a chemopreventive agent, restoring redox balance and protecting against DNA damage. In established or advanced tumors, selenium exerts cytotoxic and pro-apoptotic effects, often synergizing with chemotherapy or radiotherapy. Preclinical studies indicate that selenium pre-treatment enhances chemosensitivity by reducing oxidative stress and improving mitochondrial function, while co-administration during active therapy helps limit treatment-induced toxicity. Post-therapy supplementation may aid in recovery by replenishing antioxidant defenses and reducing the risk of recurrence. Thus, selenium’s timing in the therapeutic continuum – from prevention to adjunctive and restorative phases – should be strategically aligned with disease progression^[55]^.

### For whom? (patient-specific factors and tumor subtypes)

Selenium’s efficacy varies significantly across patient populations and breast cancer subtypes. Individuals with low baseline selenium levels – common in regions with selenium-deficient soils – derive greater benefit from supplementation than those with adequate status. Genetic polymorphisms in selenoprotein genes (e.g., *GPx1, SEP15*) can also modulate response to therapy. In terms of tumor biology, selenium shows preferential cytotoxicity in TNBC, a highly aggressive subtype characterized by high oxidative stress and absence of hormone receptors. Selenium-induced redox imbalance selectively triggers apoptosis in TNBC cells while sparing normal epithelial tissues. Furthermore, emerging evidence suggests that selenium may influence gut microbiota composition and systemic immunity, indirectly contributing to tumor suppression and improved treatment response. These observations underscore the need for personalized selenium therapy, guided by serum selenium levels, genetic background, and tumor subtype^[56]^.

## Selenium and non-apoptotic cell death pathways

Beyond its well-established role in apoptosis, selenium has emerged as a regulator of non-apoptotic cell death pathways, which play critical roles in tumor suppression, therapy response, and modulation of the tumor microenvironment. These pathways include pyroptosis, ferroptosis, and autophagy, each representing a distinct mechanism of cancer cell elimination or growth suppression^[57]^.

### Pyroptosis

Pyroptosis is an inflammatory form of programmed cell death mediated by gasdermin family proteins, typically triggered by caspase-1 activation, resulting in cell membrane pore formation and cytokine release. Recent studies suggest that selenium can enhance pyroptotic signaling in breast cancer by:

- Modulating ROS to activate inflammasomes.
- Upregulating caspase-1 activity, promoting the cleavage of gasdermin D.
- Amplifying antitumor immune responses via IL-1β and IL-18 release, which recruit cytotoxic immune cells into the tumor microenvironment.

This pro-inflammatory cell death pathway provides a complementary mechanism to apoptosis, particularly in tumors resistant to traditional apoptotic triggers^[58]^.

### Ferroptosis

Ferroptosis is an iron-dependent, lipid peroxidation-driven form of cell death that is distinct from apoptosis or necrosis. Selenium, through its incorporation into glutathione peroxidase 4 (GPx4), plays a dual role:

- At physiological levels, selenium protects cells from oxidative stress by reducing lipid peroxides, preventing ferroptosis in normal tissues.
- At therapeutic or supranutritional doses, selenium can selectively increase ROS in tumor cells, overwhelm antioxidant defenses, and sensitize cancer cells – especially TNBC – to ferroptotic cell death.

Targeting ferroptosis may be particularly valuable in breast tumors with high oxidative stress that are refractory to conventional therapies^[48]^.

### Autophagy

Autophagy is a self-degradative process that removes damaged organelles and misfolded proteins. In cancer, autophagy can be double-edged: promoting survival under stress or inducing cell death when hyperactivated. Selenium modulates autophagy by:

- Regulating mTOR and AMPK signaling, thereby influencing autophagosome formation.
- Inducing autophagic cell death in tumor cells under high oxidative stress conditions.
- Interacting with apoptosis pathways, creating a synergistic cytotoxic effect in resistant breast cancer cells^[59]^.

### Translational implications

The regulation of non-apoptotic pathways by selenium expands its therapeutic versatility. By activating pyroptosis, ferroptosis, and autophagy, selenium may overcome apoptosis-resistant phenotypes, enhance tumor immunogenicity, and improve the efficacy of combination therapies. Moreover, these pathways highlight the importance of dose optimization and patient stratification, ensuring tumor targeting without harming normal tissue^[59]^.

## Challenges in harnessing selenium for breast cancer therapy

While the therapeutic potential of selenium in breast cancer is gaining recognition, its integration into clinical practice is fraught with significant challenges. One of the foremost obstacles lies in the *narrow therapeutic window* of selenium. The element displays a dual nature – beneficial at certain levels and toxic beyond a threshold. This narrow margin complicates the formulation of universally safe dosages, especially given the interindividual variability in selenium metabolism, absorption, and excretion. The risk of selenosis, characterized by symptoms like gastrointestinal distress, hair loss, and neurological abnormalities, underscores the need for careful dose management and patient monitoring^[50]^. Another key challenge is the *heterogeneity of selenium compounds* used in research and clinical trials. Various forms, such as selenomethionine, sodium selenite, and MSA, differ in their bioavailability, metabolic pathways, and biological effects. This diversity makes it difficult to compare findings across studies or standardize treatment protocols. Furthermore, the inconsistent outcomes in clinical trials – ranging from positive anticancer effects to null or even adverse results – are often attributable to differences in selenium formulations, dosages, patient selenium status at baseline, and cancer subtypes being studied^[51]^. *Patient-specific genetic and epigenetic factors* also pose a challenge. Polymorphisms in genes encoding selenoproteins, transporters, and enzymes involved in redox regulation can influence both selenium’s efficacy and its risk profile. However, routine genetic screening for such variants is not yet standard practice in oncology settings, limiting the ability to personalize selenium therapy effectively. Additionally, the tumor microenvironment adds another layer of complexity; in some cases, selenium may suppress tumor growth, while in others, it might support cell survival by enhancing antioxidant defenses within cancer cells^[52]^.

*Translational gaps* between laboratory findings and clinical applications further hinder progress. While *in vitro* and animal studies consistently demonstrate selenium’s potential to induce apoptosis and inhibit proliferation, these results often fail to translate into meaningful clinical benefits in human subjects. This discrepancy may arise from oversimplified experimental models that do not account for human metabolic nuances, tumor heterogeneity, or immune system interactions. Bridging this gap requires more robust preclinical models, longitudinal human studies, and better stratification in clinical trials^[53]^. Another challenge is the *lack of consensus and standardized guidelines* on selenium use in cancer care. Regulatory bodies and professional associations have yet to develop unified protocols for selenium assessment, supplementation, or monitoring in breast cancer patients. This leaves clinicians navigating a gray area, relying on limited or conflicting evidence to make decisions. Moreover, public access to over-the-counter selenium supplements complicates the issue, as unsupervised use may lead to excessive intake, undermining clinical efforts and patient safety^[54].^ *Funding and research prioritization* also impact the pace of discovery. Selenium, as a naturally occurring micronutrient, lacks the commercial backing that typically fuels drug development. Pharmaceutical companies often focus on synthetic, patentable compounds with higher economic returns, leaving micronutrient research to rely on public or academic funding, which may be limited or inconsistent^[55]^. Lastly, *educational gaps* among healthcare providers and patients regarding selenium’s role in cancer biology contribute to underutilization or misuse. Many clinicians remain unfamiliar with the latest molecular insights into selenium’s mechanisms or how to interpret selenium-related laboratory tests. Simultaneously, patients may view selenium as a benign supplement, unaware of its potential interactions with chemotherapy or its context-dependent effects on tumor progression^[56,57]^.

## Selenium and non-apoptotic cell death pathways

Beyond its well-characterized ability to induce apoptosis, selenium influences several forms of non-apoptotic programmed cell death that have recently gained attention for their relevance to cancer therapy. Breast cancer cells, particularly those resistant to apoptosis-based treatments, often rely on alternative survival pathways, making non-apoptotic cell death mechanisms critical therapeutic targets. Selenium and its metabolites interact with these pathways in complex ways, highlighting new opportunities for therapeutic intervention [57].

## Selenium and ferroptosis

Ferroptosis is an iron-dependent form of regulated cell death driven by uncontrolled lipid peroxidation. Central to this process is glutathione peroxidase 4 (GPX4) – a selenoenzyme that detoxifies lipid hydroperoxides. Selenium plays a unique dual role in ferroptosis biology: it is essential for GPX4 activity, yet certain selenium compounds can paradoxically *promote* ferroptosis in cancer cells. On one hand, selenium supplementation increases GPX4 expression, supplying cells with enhanced antioxidant capacity. However, in cancer cells with already elevated oxidative stress, selenium derivatives – especially selenite and selenium nanoparticles – can disrupt glutathione (GSH) homeostasis and elevate ROS beyond repair. When GSH is depleted, GPX4 becomes functionally compromised despite selenium availability, leading to the accumulation of lipid peroxides and initiation of ferroptosis. Some studies demonstrate that methylselenocysteine accelerates lipid peroxidation and sensitizes breast cancer cells to ferroptosis inducers, suggesting that selenium’s redox-modulating properties can be strategically leveraged to overcome treatment-resistant tumors. In aggressive breast cancer subtypes, such as TNBC, where ferroptosis resistance contributes to metastatic progression, selenium-based therapies offer a promising avenue for reactivating this suppressed cell death pathway^[58]^.

## Selenium and pyroptosis

Pyroptosis is an inflammatory form of programmed cell death mediated by gasdermin proteins, typically triggered by inflammasome activation. Although pyroptosis is best known for its role in host defense, accumulating evidence shows that controlled induction of pyroptosis in tumor cells can enhance antitumor immunity. Selenium’s ability to modulate inflammatory signaling places it at a strategic position to influence pyroptotic pathways. Selenium compounds can suppress chronic inflammatory states through inhibition of NF-κB, yet in malignant cells, they may promote pyroptotic signaling by enhancing ROS production, activating caspase-1, and stimulating gasdermin D cleavage. This dual functionality allows selenium to maintain immune balance in normal tissue while selectively triggering inflammatory cell death in breast tumors. Importantly, pyroptosis releases immunogenic intracellular contents – such as HMGB1 and IL-1β – into the tumor microenvironment. Selenium-induced pyroptosis may therefore enhance antitumor immune surveillance and synergize with immunotherapies, particularly in breast cancers known to evade immune detection^[59]^.

## Selenium and necroptosis

Necroptosis represents a programmed form of necrosis driven by the RIPK1-RIPK3-MLKL axis. It often serves as a backup mechanism when apoptosis is inhibited, a common problem in chemoresistant breast cancers. Selenium compounds have demonstrated the ability to promote necroptotic signaling, especially in cells where apoptotic pathways are suppressed due to mutations in p53 or overexpression of antiapoptotic proteins. Mechanistically, selenium-induced oxidative stress can directly activate RIPK1 phosphorylation or destabilize energy metabolism, both of which promote necroptosis. Some selenium metabolites also downregulate cellular inhibitors of necroptosis while amplifying mitochondrial dysfunction – shifting cancer cells toward this alternative death pathway. This makes selenium particularly valuable in treating apoptosis-resistant tumors, where necroptosis induction can circumvent classical survival mechanisms^[60]^.

## Selenium and autophagy-dependent cell death

Autophagy is primarily a cytoprotective process that enables cells to degrade damaged organelles and adapt to metabolic stress. However, excessive or dysregulated autophagy can lead to self-digestion and cell death. Selenium exhibits a contrasting influence on autophagy depending on dose, chemical form, and cell context. At physiological doses, selenium may support autophagy-mediated stress adaptation through enhancement of antioxidant defense. In contrast, higher doses or specific selenium derivatives – such as selenocysteine, selenite, and selenium nanoparticles – can promote autophagic flux beyond survival thresholds. This occurs through:

- ROS accumulation that activates AMPK.
- Inhibition of PI3K/Akt/mTOR survival signaling.
- Increased expression of Beclin-1 and LC3-II.
- Disruption of lysosomal function.

In breast cancer models, selenium-induced autophagy has been shown to sensitize tumor cells to chemotherapy, reduce stemness, and impair metastatic potential. The ability of selenium to toggle autophagy from a survival program to a death mechanism underscores its versatility as an anticancer nutrient^[58]^.

## Selenium and parthanatos

Parthanatos is a PARP1-dependent form of cell death triggered by severe DNA damage. Selenium’s redox-modulating properties can elevate DNA oxidative lesions, leading to sustained PARP1 activation. This results in NAD⁺ depletion, AIF (apoptosis-inducing factor) translocation from mitochondria to the nucleus, and large-scale DNA fragmentation. Unlike apoptosis, parthanatos does not rely on caspase activation, making it a strategically important death pathway in apoptosis-deficient breast cancer phenotypes. Selenium’s ability to induce DNA damage selectively in malignant cells – while sparing normal tissue due to differential redox states – positions it as a valuable agent for engaging parthanatos in resistant tumors [59].

## Integration of non-apoptotic pathways in selenium-mediated therapy

The diversity of non-apoptotic cell death mechanisms engaged by selenium signals a paradigm shift in breast cancer therapy. Instead of relying solely on apoptosis, selenium compounds can simultaneously or selectively activate ferroptosis, pyroptosis, necroptosis, autophagy-dependent death, and parthanatos. This multipronged effect may:

- Enhance therapeutic efficacy.
- Overcome drug resistance.
- Generate stronger antitumor immune responses.
- Reduce tumor viability across heterogeneous cell populations^[60]^.

Such versatility makes selenium a unique candidate for combination strategies with chemotherapeutics, targeted agents, and immune-based therapies.

## Conclusion

Selenium, long recognized for its essential biological roles, has emerged as a critical player in the complex dance between cell survival and death, particularly in the context of breast cancer. The dualistic nature of this micronutrient – capable of both inducing apoptosis and restraining aberrant proliferation – positions it uniquely at the intersection of chemoprevention and therapeutic modulation. As evidence continues to mount from molecular, preclinical, and clinical studies, it becomes increasingly apparent that selenium is not just a passive dietary trace element but a dynamic modulator of oncogenic pathways, redox balance, and cell cycle control. Yet, this promising narrative is interwoven with caveats. The therapeutic efficacy of selenium is not universal but rather context dependent, shaped by its chemical form, dosage, delivery mechanism, and the unique molecular landscape of each breast cancer subtype. While certain selenium compounds demonstrate robust pro-apoptotic and antiproliferative effects in ER-negative or TNBCs, others show varied responses depending on the redox environment or the genetic profile of the tumor cells. The paradox of selenium – both essential and potentially toxic – demands a thoughtful, precision-driven approach to its application in oncology.
